# Individual and socioeconomic factors associated with childhood immunization coverage in Nigeria

**DOI:** 10.11604/pamj.2017.26.220.11453

**Published:** 2017-04-24

**Authors:** Obinna Oleribe, Vibha Kumar, Adebowale Awosika-Olumo, Simon David Taylor-Robinson

**Affiliations:** 1 Excellence and Friends Management Care Centre (EFMC), Dutse Abuja FCT, Nigeria; 2 Royal College of Physicians of London, 11 St Andrews Place, Regent’s Park, London; 3 Walden University, 100 Washington Ave S #900, Minneapolis, Minnesota, USA; 4 GHMIGROUP INC, 3845 Cypress Creek Parkway #305, Houston, Tx 77068; 5 Faculty of Medicine, Imperial College London, St Mary’s Hospital Campus, Norfolk Place, London W2 1PG, United Kingdom

**Keywords:** Immunization, Nigeria, Nigerian demographic and health survey (NDHS)

## Abstract

**Introduction:**

Immunization is the world’s most successful and cost-effective public health intervention as it prevents over 2 million deaths annually. However, over 2 million deaths still occur yearly from Vaccine preventable diseases, the majority of which occur in sub-Saharan Africa. Nigeria is a major contributor of global childhood deaths from VPDs. Till date, Nigeria still has wild polio virus in circulation. The objective of this study was to identify the individual and socioeconomic factors associated with immunization coverage in Nigeria through a secondary dataset analysis of Nigeria Demographic and Health Survey (NDHS), 2013.

**Methods:**

A quantitative analysis of the 2013 NDHS dataset was performed. Ethical approvals were obtained from Walden University IRB and the National Health Research Ethics Committee of Nigeria. The dataset was downloaded, validated for completeness and analyzed using univariate, bivariate and multivariate statistics.

**Results:**

Of 27,571 children aged 0 to 59 months, 22.1% had full vaccination, and 29% never received any vaccination. Immunization coverage was significantly associated with childbirth order, delivery place, child number, and presence or absence of a child health card. Maternal age, geographical location, education, religion, literacy, wealth index, marital status, and occupation were significantly associated with immunization coverage. Paternal education, occupation, and age were also significantly associated with coverage. Respondent's age, educational attainment and wealth index remained significantly related to immunization coverage at 95% confidence interval in multivariate analysis.

**Conclusion:**

The study highlights child, parental and socioeconomic barriers to successful immunization programs in Nigeria. These findings need urgent attention, given the re-emergence of wild poliovirus in Nigeria. An effective, efficient, sustainable, accessible, and acceptable immunization program for children should be designed, developed and undertaken in Nigeria with adequate strategies put in place to implement them.

## Introduction

Immunization remains one of the most successful and cost-effective public health interventions worldwide, preventing (and/or eradicating) several serious childhood diseases [1]. Although existing immunization programs prevent over 2 to 3 million deaths annually that could have resulted from vaccine preventable diseases (VPDs), 19.4 million children missed out on basic vaccination globally in 2015 [2]. This results in an estimated 2.7 million child deaths annually from VPDs, the majority of which occur in sub-Saharan Africa, which accounts for the increasing under-5 years-old mortality rate in sub-Saharan Africa and South Asia, as these two regions accounted for 82% of under-5 years-old deaths in 2011 [3]. The expanded program on immunization (EPI) was launched by the WHO in 1974 and has averted over 15.6 million deaths since 2000 through measles immunization, elimination of maternal and neonatal tetanus from 35 out of 59 high-risk countries, and the dramatic reduction of the prevalence of polio globally [4]. Nigeria adopted the EPI program in 1979. Despite its over 30 years of implementation in Nigeria, hundreds of millions of US dollars spent and a significant population of healthcare workers engaged in the program, the national immunization coverage is still less than 30% [5,6]. With recent wild polio virus reemergence in Nigeria, this study reanalyzed the 2013 NDHS dataset to identify and document the factors hindering acceptable immunization coverage levels in Nigeria [2, 7, 8]. The study was designed to answer the following research questions: (A) is there an association between socioeconomic factors (education and income level) and percentage of completeness of immunization for Nigerian children, and (B) is there an association between individual factors (child's gender and birth order) and percentage of completeness of childhood immunization in Nigeria, using the health belief model (HBM) and the social ecological model (SEM) theoretical framework.

## Methods

The Demographic and Health Survey (DHS) is a program funded by United States Agency for International Development (USAID). The last DHS study was conducted in Nigeria in 2013 and the dataset is managed by USAID under the DHS Program. We reanalyzed the Nigerian DHS 2013 dataset. The data used were collected from the entire country and representative samples were collected from each enumeration area (EA) at a fixed sample of 45 per EA. The survey was implemented by the National Population Commission of Nigeria from February 2013 to June 2013. 38,522 households were sampled, from which 38,948 females and 17,359 males aged 15 to 49 years were interviewed [9]. All women aged 15 to 49 who were either permanent residents or visitors to the selected households, and had children aged 12 to 24 months were included and interviewed by trained health workers. Permission to download and reanalyze the data was sought and obtained from dataset holders. The dataset was analyzed using SPSS^®^ Version 21 [10]. Initial descriptive analyses were done to check for outliers, missing data, and consistency of the data set. Univariate, bivariate and multivariate analyses were done with completed immunization as the only dependent variable. Ethical approvals were received from Walden University Institutional Review Board (IRB) and National Health Research Ethics Committee of Nigeria (NHREC).

**Dataset manipulation**: The childhood immunization coverage level was the dependent variable in this study. Completed immunization refers to any child who has had the six vaccine preventable disease vaccines of Bacille-Calmette Guerin (BCG), third dose of diphtheria, pertussis and tetanus (DPT3), third dose of oral polio vaccine (OPV3,) and measles by 24 months. Any child who received fewer vaccines (less than three OPVs [minus OPV0], less than 3 DPT, no BGC and/or measles) was classified as partial recipients. Any child with all except OPV0 was also classified as complete and protected. Anybody without DPT1 and OPV1 was classified as without immunization, irrespective of whether the child had OPV or measles vaccination from national campaign programs. A score of 4 (BCG = 1, DPT3 = 1, OPV3 = 1, and Measles = 1) meant complete immunization while anything less than this was seen as incomplete immunization. DPT3, BCG, OPV3 and measles were combined to compute a new variable named 'Completed Immunization'. Children who had none were classified as having not received vaccination-even if they had OPV0, 1 or 2; or DPT 1 and 2. This was further recoded to have children who had received the four vaccines as only those who completed vaccination. The key independent variables in this study were individual and socioeconomic factors of the participants in the NDHS 2013 study. These include age, marital status, highest education level, husband's/partner's education attainment, literacy, wealth index, the respondent having worked in the last 12 months, and the respondent's occupation [11]. Child-related independent variables were child birth order and child gender. These variables were dichotomized for logistic regression analysis. For instance, parental age was re-classified into less than 30 years and above 30 years, marital status was reclassified into married and single, with single including single, divorced, widowed, or separated. Similarly, educational level was reclassified into having West African Examination Council/General Certificate of Education (WAEC/GCE) or not having WAEC/GCE, and employment status was reclassified into employed with salary, or not employed (including employed without pay).

In the ''received vaccination'' group, all those who stated they did not have the vaccination were grouped along with those responded ''don't know'' with the assumption that mothers were unlikely to ever forget immunizing their children. Furthermore, all those who said their children were vaccinated were grouped together whether it was documented in their cards or not. In analyzing for vaccination coverage, all children aged 0 to 59 months (= 0, <60) were selected and analyzed. Respondents' age was recoded into < 30 and ≥30 years, the region of origin into North (North Central, North East and North West) and South (South-South, South East and South West), education into not educated (none or did not complete primary) or educated (completed primary and above), religion (Islam and others), literacy (can read and cannot read), wealth (poor and not poor), number of children (< 3 and ≥ 3), marital status (married and not married), profession (not working, professional/skilled manual and others), place of birth (home, public and private), health card (had one or did not have any), and vaccinated (was vaccinated and was not vaccinated). Respondents' spouse/partners' information were also similarly recoded. This was to allow for a dichotomous analysis of the findings. The key maternal (highest educational level, education attainment, wealth index, literacy), husband/partner (educational attainment and highest educational level) and child factors (child index, number in family, delivery place and availability of health card) were further manipulated for linear regression (bivariate) analysis. Their Z-scores were calculated and a sum of all maternal, husband/partner and child factors developed. A linear regression of each set of factors was then developed.

**Data analysis**: We revalidated the data set using built in validation functions in SPSS^®^ V21 [10]. We then conducted simple descriptive analyses. To ensure effective data analysis, we recoded identified variables, categorized and manipulated them in line with the research questions and data operationalization plans. From the descriptive analysis, we developed simple tables, charts and graphs to describe the dataset. Univariate (simple frequencies distributions, bar charts, line graphs and pie charts), bivariate (correlation coefficients, cross tables, Chi square and simple linear regression) and multivariate (logistic regression) analyses were performed to identify associations and measure levels of significance between independent and dependent variables [12]. We calculated the correlation coefficient (r), alpha values and confidence intervals [12]. Finally, we performed multiple logistic and linear regression analyses to reduce statistical errors [13].

## Results

A total of 31,482 persons who responded to the survey had children within the age range of 0 to 5 years, with an average age of 29.46 ± 7.0 years, and a modal age range of 30 years. A total of 31,482 persons who responded to the survey had children within the age of 0 to 5 years, with an average age of 29.46 ± 7.0 years, and a modal age of 30 years.

**Figure 1:**
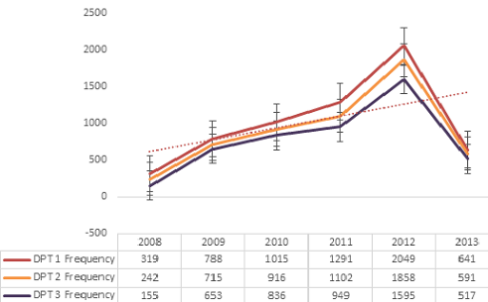
Trend of DPT1 – 3 from 2008 through 2013 in NDHS, 2013 survey

**Figure 2:**
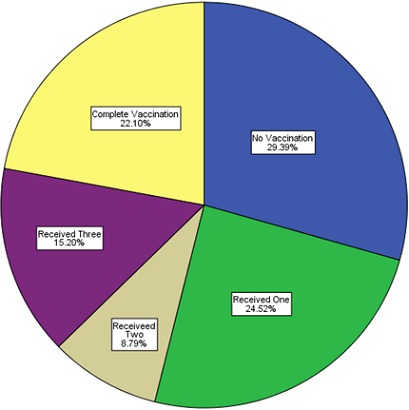
Degree of completeness of vaccination of children in the Nigerian DHS study population

**Univariate analysis**: The sociodemographic details are as depicted in Table 1. All were or had ever been married, about one-third (10352, 31.9%) resided in urban regions of their respective states, 17.7% (5596) started primary (6.2) and secondary (11.5%), but did not complete their education, while only 19% (5991) had 6 or more children in their households. Among the husbands/partners, 85% (23,432) were 30 years or older, 65.8% (18.138) were either professionals or involved in skilled manual work, and 39.9% (10,991) were unable to read as shown in Table 2. A total of 27,571 children were within the age of 0 to 59 months with an average age of 28.01 ± 17.31 months, (median = 27 months; mode = 13 months), and 50.71% (15,965) were males. Over 61.8% (17,026) of the children were delivered at home and 48.3% (13,311) had no health cards for their children. While 51.3% (14,155) of the children were within the first three in birth order, more than 75% of all children were born within the first five in birth order. A large number of the children were not properly immunized as about 29% (5881) had never received vaccination before, and more than 48% (13,255) did not receive the BCG vaccination; and the absolute number of people vaccinated increased gradually over the years from 2008 to 2012, but suffered a major decline in 2013 as shown in Table 3 and Figure 1. BCG, OPV (1-3) and measles exhibited a similar trend as DPT (1-3) shown in Figure 1. Only 22.1% of all respondents completed vaccination (BCG+OPV3+DPT3+Measles) while 29% did not have any vaccinations at all Figure 2.

**Table 1:** Sociodemographic details of respondents in the study, NDHS, 2013

Age of respondents	Frequency	Percent	Cumulative percent
15-19	1531	4.9	4.9
20-24	6083	19.3	24.2
25-29	8762	27.8	52
30-34	6936	22	74
35-39	4923	15.6	89.7
40-44	2344	7.4	97.1
45-49	903	2.9	100
**Region of respondents**			
North-Central	4614	14.7	14.7
North-East	6517	20.7	35.4
North-West	9906	31.5	66.8
South-East	2816	8.9	75.8
South-South	3747	11.9	87.7
South-West	3882	12.3	100
**Educational level**			
No education	14762	46.9	46.9
Primary	6432	20.4	67.3
Secondary	8365	26.6	93.9
Higher	1923	6.1	100
**Religion**			
Catholic	2540	8.1	8.1
Other Christians (other than Catholics)	10114	32.1	40.4
Islam	18354	58.3	99
Traditionalist	302	1	100
Other	12	0	100
Total	31322	99.5	
Missing	160	0.5	
**Literacy level**			
Cannot read at all	18153	57.7	57.9
Able to read only parts of sentence	2165	6.9	64.8
Able to read whole sentence	10879	34.6	99.6
No card with required language	128	0.4	100
Blind/visually impaired	10	0	100
**Wealth index**			
Poorest	7076	22.5	22.5
Poorer	7386	23.5	45.9
Middle	6272	19.9	65.9
Richer	5806	18.4	84.3
Richest	4942	15.7	100

**Table 2:** A comparison between respondents and respondent’s spouse/partners educational level and occupation

Educational level	Respondents (%)	Husband/partners (5)
No education	46.9	36.9
Primary	20.4	19
Secondary	26.6	28.6
Higher	6.1	12.6
**Occupation**		
Did not work	28.9	0.9
Professional/technical/managerial	3.6	11.6
Clerical	0.3	0.8
Sales	38	18.6
Agricultural - self employed	0.8	4.5
Agricultural - employee	11.2	31.3
Services	4.7	5.5
Skilled manual	11.5	19.5
Unskilled manual	0	4.7
Other	0.1	0

**Table 3:** Vaccination among children aged 0 to 59 months in 2013 NDHS survey

	BCG*		DPT3**		Polio 3***	Measles		
Description	Frequency	Percent	Frequency	Percent	Frequency	Percent	Frequency	Percent
No	13255	48.1	17301	**62.8**	13833	50.2	16899	61.3
Vaccination date on card	6603	23.9	4769	17.3	4599	16.7	3785	13.7
Reported by mother	7541	27.4	5242	19	8484	30.8	6580	23.9
Vaccination marked on card	87	0.3	98	0.4	131	0.5	120	0.4
Don't know	56	0.2	102	0.4	26	0.1	69	0.3
**Total**	27542	99.9	27512	99.8	27073	98.2	27453	99.6

* The bacille-calmette guerin (BCG) vaccine ** Third dose of diphtheria-tetanus-pertussis (DTP3)***Third dose of oral polio vaccine

**Bivariate and multivariate analysis**: In bivariate analysis, respondents’ age, place of residence, religion, education, literacy, wealth index, occupation, and marital status were all significantly related to immunization coverage and completion of immunization, as shown in Table 4, Table 5. Similarly, the respondents' partner/husbands age, occupation and education were all significantly related to immunization coverage. While child birth order, the number in the family, the delivery place, and the presence or absence of a child health card were all significantly related to immunization coverage, the sex of the child was not. All the above factors were therefore significantly related to immunization coverage rates in 2013 in Nigeria. Marital state of respondents and sex of the child were not significant in binary logistic analysis. Correlation coefficients were computed among the various independent variables with the dependent variables. Correlations coefficient with control for Type 1 error using the Bonferroni correction showed that high literacy, better wealth index, and residing in the southern part of Nigeria were significantly related to improved vaccination rate as shown in Table 6. Similarly, only high husband/partner's literacy; the child's place of delivery, the presence of a health card, and the number of children in the family significantly related to improved vaccination rate from correlational studies. Using a five level concept for the complete vaccination process, correlation between the complete immunization and region (r=0.38); educational attainment (r=0.47); religion (r=0.39); literacy (r=0.46); and wealth index (r=0.42); displayed statistical significance (p.001). We went on to perform linear (bivariate and multivariate) regression analysis to evaluate the completion of immunization from respondents, husband/partners' and child factors using a random-effects model [11]. The 95% confidence interval for the slope shows that the respondent, husband/partner and child overall relationship was significantly related to the immunization coverage. However, the accuracy in predicting the immunization coverage was moderate, as can be seen in Table 7. Finally, we undertook a multiple regression analysis to evaluate how well the factors of interest predicted immunization coverage in children. The linear combination of maternal, paternal and child variables were significantly related to immunization coverage, maternal R2 = 0.3; husband/partner R2 = 0.19; and child R2 = 0.39. The sample multiple correlation coefficient was 0.53 (maternal), 0.51 (husband/partner) and 0.37 (child). The respondents' age, educational attainment and wealth index at 95% confidence interval showed a significant relationship between these variables and immunization coverage of children Table 6, Table 7.

**Table 4:** Respondants’ personal and social economic factors influence on vaccination coverage in DHS 2013 survey

Vaccination	Chi Squared (X^2^)	d.f	Test (2-sided)	Eta
**Age**				
BCG	108.2	1	0.00	0.063
DPT 3	173.5	1	0.00	0.079
OPV 3	40.7	1	0.00	0.039
Measles	236.5	1	0.00	0.093
**Region**				
BCG	5350.5	1	0.00	0.441
DPT 3	4531.5	1	0.00	0.406
OPV 3	37.8	1	0.00	0.037
Measles	2640.5	1	0.00	0.31
**Education**				
BCG	7859.8	1	0.00	0.534
DPT 3	6043.8	1	0.00	0.469
OPV 3	222.2	1	0.00	0.091
Measles	4200.5	1	0.00	0.391
**Religion**				
BCG	5704.3	1	0.00	0.456
DPT 3	4474.8	1	0.00	0.404
OPV 3	55.1	1	0.00	0.045
Measles	2948.2	1	0.00	0.329
**Literacy**				
BCG	7398.8	1	0.00	0.519
DPT 3	5788.1	1	0.00	0.46
OPV 3	289.3	1	0.00	0.104
Measles	3915.6	1	0.00	0.379
**Wealth index**				
BCG	6370	1	0.00	0.481
DPT 3	4981.8	1	0.00	0.426
OPV 3	104.9	1	0.00	0.62
Measles	3242.4	1	0.00	0.344
**Marital status**				
BCG	210.6	1	0.00	0.519
DPT 3	116.4	1	0.00	0.46
OPV 3	8.5	1	0.00	0.104
Measles	76.5	1	0.00	0.379
**Occupation**				
BCG	669.2	2	0.00	0.137
DPT 3	540	2	0.00	0.127
OPV 3	171.7	2	0.00	0.075
Measles	605.7	2	0.00	0.128

**Table 5:** Individual and socioeconomic factors on completed immunization rate, 2013

Description	Chi squared	d.f*	Test**	Eta
**Respondents'**				
Age	213.9	4	0.00	0.087
Region	4338.4	4	0.00	0.378
Education	6351	4	0.00	0.468
Religion	4534.2	4	0.00	0.388
Literacy	60.98.7	4	0.00	0.461
Wealth Index	5016	4	0.00	0.451
Marital Status	160	4	0.00	0.071
Occupation	860.8	8	0.00	0.169
**Respondents' husbands/partners**				
Age	55	4	0.00	0.041
Occupation	1328.8	8	0.00	0.214
Education	5017	4	0.00	0.413
**Respondents' children**				
Birth Column	3.36	4	0.00	.000
Birth Order	322.6	4	0.00	0.102
Number of Children	193.4	4	0.00	0.078
Delivery Place	5922.7	8	0.00	0.449
Health Card	13460.6	4	0.00	0.69

*Degree of freedom; **Test of significance

**Table 6:** Pearson correlation for respondents to the NDHS 2013 survey

	BCG	DPT 3	OPV 3	Measles
Age	0.063^**^	0.079^**^	0.039^**^	0.093^**^
Region of Respondents	0.441^**^	0.406^**^	0.037^**^	0.310^**^
Education	0.534^**^	0.469^**^	0.091^**^	0.391^**^
Religion	-0.456^**^	-0.404^**^	-0.045^**^	-0.329^**^
Literacy	0.519^**^	0.460^**^	0.104^**^	0.379^**^
Wealth	0.481^**^	0.426^**^	0.062^**^	0.344^**^
Marital Status	-0.087^**^	-0.065^**^	-0.018^**^	-0.053^**^
Occupation	0.137^**^	0.127^**^	0.075^**^	0.128^**^

**Significant findings

**Table 7:** Bivariate analysis of respondents, husband/partners and child-related factors

			Sig.	95.0% Confidence Interval for B		
Model	B	Std. Error			Lower bound	Upper bound
Respondents factors	0.221	0.002	103.348	0.00	0.217	0.225
Husband/partners factors	0.433	0.007	60.742	0.00	0.419	0.447
Child factors	0.347	0.004	82.860	0.00	0.339	0.355

## Discussion

In this Nigerian study, less than 23% of targetable children received complete immunization and close to one third of the children did not receive any vaccination at all. This is similar to the findings of Obiajunwa and Olaogun in 2013 in South-Western Nigeria where they recorded 26.5% coverage in a region which was expected to have very high immunization coverage [14]. In Ethiopia, Lakew et al. (2015) found that there was just 24.3 % full immunization coverage [15]. It is also similar to the WHO's assertion in 2015 that 1 out of every 5 children still miss routine immunizations [8]. This means that Nigeria, with an under-5 year population of about 30,546,274 as of 2013 [16], contributed over 7.5 million (34%) children to the global 22.4 million non-immunized pool. This increases the risk of vaccine preventable diseases among under 5-year-old children [17] and may explain why Nigeria is one of the six nations in the world with the worst under-5 years-old mortality rate (117.4/1000), contributing 11% of the total global mortality rate [18,19]. With the reemergence of the wild polio virus in Nigeria after its near elimination, Nigeria and Nigerians have a lot of work towards ensuring the elimination of polio and other vaccine preventable diseases and deaths in Nigeria [8,20]. The 29% of qualified children who did not receive any vaccination at all is significantly higher than the 11.9% documented by Obiajunwa and Olaogun (2013) in the South-Western Nigeria [14]. Among the non-vaccinated, more than 48% did not receive BCG vaccination, and over 50% of the children did not receive DPT 1, 2, and 3. The 55% of children who did not receive the 'Polio 0' vaccine may be a reflection of the high level of home delivery in Nigeria, estimated to be 40% to 45% [21].

**Child factors**: Child-related factors, such as number of children in the household, the place of delivery, the child birth order, and the presence or absence of a child health card affected immunization coverage. Correlations also showed that child factors, such as the place of delivery, the presence of a health card, and the number of children in the family were significantly related to vaccination rate. These findings are in agreement with previous findings in western Kenya, where better knowledge of vaccination schedules, longer birth interval/first birth, fewer number of children under-5 in a household, and interaction between literacy and wealth were found to be significantly associated with complete vaccination [22]. However, the sex of the child was found not to be significant in determining immunization coverage in Nigeria (p > 0.05). Who attended to the birth of a child (similar to delivery place) had previously been found to affect immunization coverage in Lao People´s Democratic Republic [23]. A similar finding was also documented by Fatiregun and Okoro in 2012 in a previous Nigerian study [24]. In another study in Ethiopia, researchers discovered that having a vaccination card improved the chances of immunization coverage [15]. However, how immunization card presence affects coverage is unknown and required further qualitative or mixed studies.

**Maternal factors**: In previous studies, maternal factors have been found to impact childhood immunization coverage [14,23,25]. In this study, statistical analysis revealed that maternal age, region, religion, education status/literacy level, wealth index, marital status, and occupation all directly affected commencement, continuation, and completion of immunization. Multivariate analysis showed that high literacy, better wealth index, and residing in the southern part of the country were significantly related to better vaccination rates. This finding supports the findings of Kitamura et al. (2013) and Danis et al. (2010) that continuation and completion of the required number of vaccinations in children depended on the mother´s educational level, socioeconomic status, employment status, immigration status, race, experience with vaccination services, health insurance, parental beliefs, attitudes towards immunization, and adequate schedule information [23, 25]. Although not all the variables studied by Kitamura et al. (2013) and Danis et al. (2010) were studied in this work, maternal factors were clearly shown to affect commencement, continuation, and completion of the required number of immunizations as maternal age, education, and occupation were still statistically significant factors affecting immunization coverage in multivariate analysis [23,25]. Moreover, geographical location (i.e. region) was previously documented to influence immunization coverage by Doctor et al. (2011) in northern Nigeria, with people living in urban areas having usually higher coverage rate [26]. This may be a result of better access, a good transportation system, higher rates of literacy, and a better wealth index. This was not, however, studied in this current work as the dataset analyzed did not differentiate respondents based on urban or rural locations. The influence of religion as a factor was also documented by Ophori et al. (2014) [27]. Furthermore, the findings of Lakew et al. (2015) in Ethiopia are similar to this as they discovered that full immunization coverage was commoner among women in rich wealth index groups [15].

**Paternal factors**: From literature reviews, the least studied variables concerning immunization coverage are paternal factors. We discovered that paternal age, occupation, and educational status directly affected immunization coverage. Our results suggested that the husband's high literacy is significantly related to improved vaccination rate. The study corroborates findings by Danis et al., (2010) that paternal education level directly affects the rate of immunization of children within the household [24]. The study validates findings by Antai (2012) from a high level multivariate analysis of the same dataset [28].

**Future research work**: As the current secondary data analysis did not exhaustively explore the factors that may be responsible for immunization coverage in Nigeria, as parents and key stakeholders were not interviewed using a tailored data collection tool, more in-depth interviews and focus group discussions are recommended to identify the root cause(s) of the low coverage. Studies involving both primary quantitative and qualitative data collection by the researcher are recommended. Future studies should look at other relevant variables, such as rural/urban, health system, community-related and policy/governance factors which the current study did not explore. Findings from these additional studies should be used to develop tailored mechanisms and processes to improve immunization coverage rate to eliminate VPDs in Nigeria.

## Conclusion

The findings from this study revealed that: (A) there is an association between parental socioeconomic factors (education and income level) and (B) child individual factors (child's gender and birth order) and percentage completeness of childhood immunization in Nigeria. As immunization has remained the most effective and efficient public health intervention to date, the Nigerian Government should restructure the process to improve vaccination uptake and reduce childhood morbidity and mortality. New policies based on identified individual and socioeconomic factors should be developed, and implemented to improve child survival indices in Nigeria. That Nigeria contributes over 25% of global childhood deaths, that over 50% of Nigerian parents are ignorant of vaccination schedules, that over 45% live below the poverty level, that only 23% of Nigerian children are fully protected against VPDs, and that WPV still exists in Nigeria are all unacceptable issues. Identified major parental and child-related factors hindering immunization coverage are preventable with the right political will, proper funding and social mobilization. We should institutionalize routine immunization to save Nigerian children from avoidable VPDs, and untimely deaths. We call on all stakeholders to safeguard the health of Nigerian children. Immunization should be made compulsory for all children. Parents should be supported to access these services. Health insurance should be provided to reduce out-of-pocket expenditure and community support should be galvanized to ensure that every child is fully immunized. Finally, mentors should be developed for families with high birth order to ensure that no child fails to receive vaccination. The re-emergence of WPV in Nigeria should provide the motivation needed to make the necessary changes in the healthcare industry.

### What is known about this topic

- Immunization is the most effective and efficient public health intervention known to man;
- Although, 2 to 3 million children have been saved by immunization in recent years, over 2,000,000 children still die yearly from vaccine-preventable diseases;
- Nigeria remains a major contributor to global VPD deaths, habitat for polio virus and a rate determining step in the fight to eradicate polio from the world.

### What this study adds

- Nigeria immunization coverage rate is low - less than 25% completion rate. Thus, several avoidable childhood deaths occur from vaccine-preventable diseases (VPD);
- This study identified statistically significant child, maternal and paternal factors that hinder the achievement of projected immunization coverage rates in Nigeria;
- These factors must be addressed for Nigeria to make meaningful progress towards the elimination of vaccine preventable diseases in the country.
